# Spider Silk‐Inspired Artificial Fibers

**DOI:** 10.1002/advs.202103965

**Published:** 2021-12-19

**Authors:** Jiatian Li, Sitong Li, Jiayi Huang, Abdul Qadeer Khan, Baigang An, Xiang Zhou, Zunfeng Liu, Meifang Zhu

**Affiliations:** ^1^ State Key Laboratory of Medicinal Chemical Biology College of Pharmacy and College of Chemistry Key Laboratory of Functional Polymer Materials Frontiers Science Center for New Organic Matter Nankai University Tianjin 300071 China; ^2^ School of Chemical Engineering University of Science and Technology Liaoning Anshan 114051 China; ^3^ Department of Science China Pharmaceutical University Nanjing 211198 China; ^4^ State Key Laboratory for Modification of Chemical Fibers and Polymer Materials College of Materials Science and Engineering Donghua University Shanghai 201620 China

**Keywords:** biomimetics, hydrogel fiber, recombinant spider silk, spider silk, strong and tough fibers

## Abstract

Spider silk is a natural polymeric fiber with high tensile strength, toughness, and has distinct thermal, optical, and biocompatible properties. The mechanical properties of spider silk are ascribed to its hierarchical structure, including primary and secondary structures of the spidroins (spider silk proteins), the nanofibril, the “core–shell”, and the “nano‐fishnet” structures. In addition, spider silk also exhibits remarkable properties regarding humidity/water response, water collection, light transmission, thermal conductance, and shape‐memory effect. This motivates researchers to prepare artificial functional fibers mimicking spider silk. In this review, the authors summarize the study of the structure and properties of natural spider silk, and the biomimetic preparation of artificial fibers from different types of molecules and polymers by taking some examples of artificial fibers exhibiting these interesting properties. In conclusion, biomimetic studies have yielded several noteworthy findings in artificial fibers with different functions, and this review aims to provide indications for biomimetic studies of functional fibers that approach and exceed the properties of natural spider silk.

## Graphical abstract

The authors summarize the study of the structure and properties of natural spider silk, and the biomimetic preparation of artificial fibers from different types of materials by taking some examples of artificial fibers exhibiting these interesting properties. This review aims to provide indications for biomimetic studies of functional fibers that approach and exceed the properties of natural spider silk.

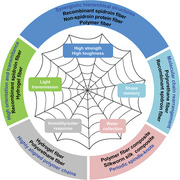

## 1 Introduction

Spiders are one of the oldest species on Earth and have evolved over the course of hundreds of millions of years. Spider silk is vital to spiders for catching prey and escaping from predators. Some researchers have discovered that spiders can spin a single thread of length approximately up to 25 m, spanning across a river in Madagascar.^[^ ^1^ ^]^ Spider silk is a natural biofiber, and has attracted extensive research because of its extraordinary properties including high tensile strength and toughness,^[^ ^2^ ^]^ high thermal conductivity,^[^ ^3^ ^]^ supercontraction,^[^ ^4^ ^]^ and peculiar torsion rotational actuation.^[^ ^5^ ^]^ These excellent properties make spider silk an attractive biological material for potential applications in artificial ligaments, heat sinks, artificial tendons, artificial muscles, bulletproof vests, parachutes, etc.

The wonderful combination of high strength and high toughness in spider silk is inextricably linked with the unique hierarchical structures, such as the amino acid sequence and secondary and tertiary structures of spidroins.^[^ ^6^ ^]^ Spidroins are high‐molecular‐weight proteins (typically 250–400 kDa) with variable sequences, depending on the silk type and the spider species.^[^ ^7^ ^]^ Spidroins usually consist of a highly repetitive sequence segment flanked by amino‐ and carboxyl‐terminal domains (NTD and CTD, respectively), and their primary structures are unique for different silk types and spider species.^[^ ^8^ ^]^ It was observed that the sequence and the secondary structures (such as *β*‐sheet and *α*‐helix) are highly correlated with their specific mechanical properties.^[^ ^9^ ^]^ A comprehensive study of the structure–property relationship is ongoing for the diverse sequences and sub‐structures of different types of spidroins.

**Figure 1:**
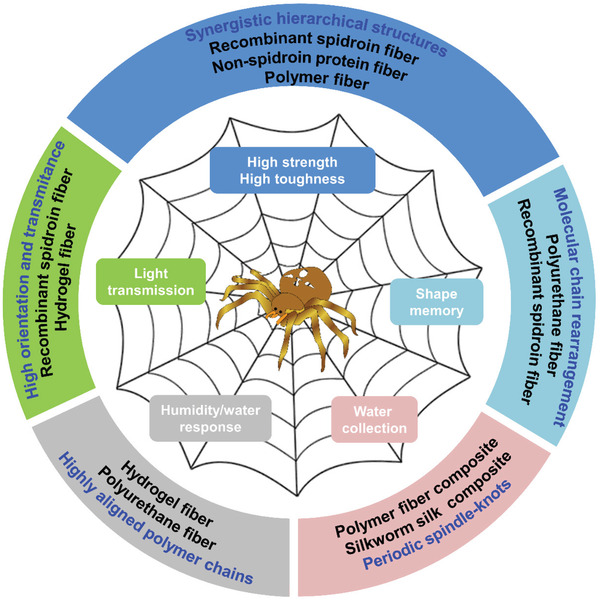
Properties of spider silk and the inspired artificial fibers. The spider drawing is adapted from http://cliparts.co/vector‐spider‐web.

Understanding the structure of spider silk can help explain the exceptional properties of spider silk, thereby providing a theoretical basis for artificially preparing spider silk‐like fibers. Strong and tough fibers can be used in cables, constructions, parachutes, bulletproof vests, and in civilian applications of textiles and fiber‐reinforced composites for energy absorption and conservation. In this review, we first discuss the structure and properties of natural spider silk, and the progress in the biomimetic preparation of artificial fibers with high strength and toughness, employing recombinant spidroins, non‐spidroin proteins, polypeptides, and polymer materials (**Figure** **1**). We further discuss the potential problems that need to be addressed and the perspectives required in future studies.

## 2 Strong and Tough Fibers by Mimicking Spider Silk

### 2.1 Mechanical Properties and the Spidroin Structure

Spider silk is famous for its high Young's modulus, tensile strength, and toughness. Spider dragline silk shows higher tensile strength and extensibility than silkworm silk. It is also tougher than most man‐made fibers such as nylon 6, 6; Kevlar; and carbon fibers. Different types of spider silks evolved to have different functions and mechanical properties, and their synergy greatly aids the survival of spiders. Orb‐weaving spiders have many glands that produce seven types of silk, each with different functional and mechanical properties, used for different purposes, including predation, hiding, walking, and egg wrapping.^[^ ^10^ ^]^ The seven types of spider silks are named after their respective secreting glands: major ampullate (MA), minor ampullate (Mi), flagelliform (Flag), aggregate, pyriform, aciniform (Ac), and cylindriform (Cyl) glands.^[^ ^10a^ ^]^ Their mechanical properties are summarized in **Table** **1**, in comparison with other common fibers.

**Table 1:** The mechanical properties of different types of spider silk and some commonly used fibers

Materials	Strength [MPa]	Strain [%]	Toughness [MJ m^−3^]	Young's modulus [GPa]	Ref.
MA silk (*A. sericatus*)	710	24.0	106	8.6	[11]
MA silk (*A. diadematus*)	824	40.0	194	1.2	[11]
MA silk (*A. trifasciata)*	1137	21.5	115	9.2	[1]
MA silk (A. argentata)	1217	22.8	136	8.0	[11]
MA silk (*D. spinose*)	1345	19.1	124	10.4	[1]
MA silk (*L. venusta*)	1469	23.3	151	10.6	[1]
MA silk (*C. darwini*)	1652	52.0	354	11.5	[1]
Flag silk (*A. argentata*)	95	465	75	0.001	[11]
Flag silk (*N. clavipes*)	142	617	27	–	[12]
Flag silk (*A. diadematus*)	233	475	283	–	[11]
Flag silk (*A. sericatus*)	296	329	150	–	[11]
Flag silk (*N. oaxacensis*)	510.8	462	92	–	[12]
Mi silk (*A. argentata*)	669	40.1	137	10.6	[11]
Mi silk (*A. sericatus*)	483	55.6	150	8.9	[11]
Ac silk (*A. argentata*)	636	50.5	230	10.4	[11]
Ac silk (*A. trifasciata*)	687	83.0	376	9.8	[11]
Cyl silk (*A. argentata*)	360	33.7	95	11.6	[11]
Cyl silk (*A. diadematus*)	270	32.0	–	8.7	[11]
*B. mori* silk	500–600	15.0–18.0	70–80	7	[13]
Mussel byssus (Distal)	75	109	45	0.87	[14]
Mussel byssus (Proximal)	35	200	35	0.02	[14]
Elastin	2	150	1.6	0.0011	[14]
Tendon collagen	150	12	7.5	1.5	[13a]
Kevlar 49	3600	2.7	50	130	[13a]
Nylon 66	750–950	–	80	2–3.6	[15]
Carbon fiber	4000	1.3	25	300	[13a]
High‐tensile steel	1500	0.8	6	200	[14]

The MA glands secrete dragline silk (also called lifeline) that is used by spiders to escape predators, and also employed as frame threads and web radii.^[^ ^16^ ^]^ The tensile strength of dragline silk is higher than that of other types of spider silk. For example, the tensile strength of the MA silk from the spider *A. argentata* was 1217 MPa, which was three times higher than that of the Cyl silk. The MA silk from *C. darwini* showed an even higher tensile strength of 1652 MPa and toughness of 354 MJ m^−3^.^[^ ^15^ ^]^

**Figure 2:**
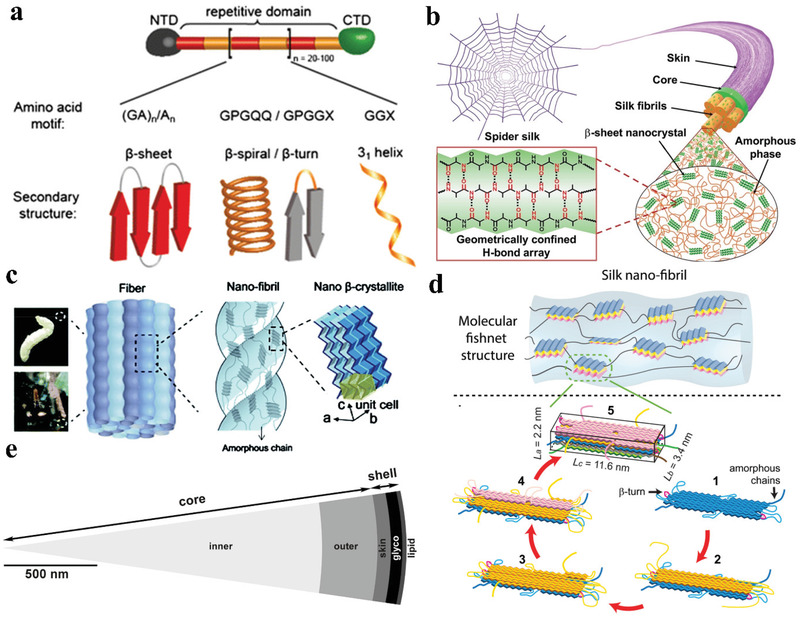
a) Schematic structure of MA spidroins including repetitive amino acid motifs and the corresponding secondary structures. Reproduced with permission.^[^ ^51^ ^]^ Copyright 2015, Springer Nature. b) Schematic illustration of the hierarchical structure of the spider silk. Reproduced with permission.^[^ ^91^ ^]^ Copyright 2021, Wiley‐VCH. c) Both the silkworm silk and the spider dragline silk are composed of interlocking nanofibrils, where *β*‐crystallites are connected by the amorphous chains and form a network. Reproduced with permission.^[^ ^30^ ^]^ Copyright 2014, Royal Society of Chemistry. d) The schematic “fishing‐net” structure of the spider silk. The *β*‐crystals are formed by layer‐by‐layer stacking of the *β*‐sheets on the initial *β*‐sheet, which are formed by folding of the repetitive protein molecular chains serving as a crystal nucleation seed. Reproduced with permission.^[^ ^35^ ^]^ Copyright 2016, Wiley‐VCH. e) The multilayer structure of the spider dragline silk. Reproduced under the terms of the Creative Commons Attribution License.^[^ ^38^ ^]^ Copyright 2007, The Authors. Published by PLOS.

Spider dragline silk is composed of spidroins, including the major ampullate spidroin 1 (MaSp1) and the major ampullate spidroin 2 (MaSp2),^[^ ^17^ ^]^ both containing crystalline regions of poly(alanine). The amorphous part of MaSp1 is a glycine‐rich sequence dominated by tripeptides (GGA and G*X*G, where *X* = Q, Y, L, or R). The amorphous part of MaSp2 is a proline‐rich sequence dominated by pentapeptides (GPGQQ and GPGGY).^[^ ^18^ ^]^ The aforementioned types of spidroins consist of highly repetitive core domains flanked by nonrepetitive NTDs and CTDs (**Figure** **2**a). The repetitive core domains contribute to the mechanical strength by forming *β*‐sheet nanocrystals, and the NTDs and CTDs contribute to fiber formation.^[^ ^19^ ^]^ Upon fiber assembly, the size and distribution of the secondary structure elements of the spidroins also contribute to their mechanical properties. During spinning, some random coils and/or helical structures of the spidroins in the spinning dope transform into *β*‐sheets via extrusion and shear flow.^[^ ^20^ ^]^ The *β*‐sheets aggregate to form crystalline regions embedded in the amorphous matrix, serving as crosslinks and contributing to the mechanical strength (Figure 2b), while the amorphous regions contribute to the elasticity.^[^ ^21^ ^]^ The mechanical properties of the dragline silk, which is forcibly spun from spiders, are related to the spinning speed.^[^ ^4^, ^22^ ^]^ A high spinning speed results in a high initial Young's modulus and fracture strength with a decreased breaking strain. Therefore, the spinning speed should be controlled uniformly to obtain spider silk with uniform mechanical properties.

Flag silk constitutes the core outline of the web spiral, and is the most extendable (reaching 617% strain) among the different types of spider silks.^[^ ^23^ ^]^ This high extensibility of the Flag silk originates from the proline‐rich repetitive domains (GPGG*X*), capable of absorbing a large amount of impact energy of the flying prey.^[^ ^24^ ^]^ The aggregate silk is sticky and covers the flag silk, serving as the glue that prevents the prey from escaping.^[^ ^25^ ^]^ The Mi silk exhibits a tensile strength approximately half that of the MA silk, which constructs the central spiral region of the spider web and stabilizes the web. The Mi and the MA spidroins mainly contain repeating units of poly(glycine‐alanine) and poly(alanine), respectively.^[^ ^26^ ^]^ This difference is considered to be the root cause behind the absence of supercontraction behavior of the Mi silk in response to water.^[^ ^27^ ^]^ The pyriform silk provides attachment points for dragline silk to adhere to trees or buildings. The Ac silk exhibits the highest toughness among different types of spider silk (376 MJ m^−3^) and is employed to wrap the prey.^[^ ^28^ ^]^ Cly silk is used in building the outer layer of the spider's egg case for warmth and protection.

### 2.2 Other Models Contributing to the Mechanical Properties of the Spider Silk

#### 2.2.1 Nanofibrillar Structure

Nanofibrils, a highly ordered architecture, commonly play an important role in deciding the mechanical properties of biological materials, such as spider silk.^[^ ^29^ ^]^ Figure 2c illustrates the hierarchical structures of spider dragline silk and silkworm silk.^[^ ^30^ ^]^ Atomic force microscopy (AFM) revealed that both the spider dragline and silkworm silk consist of bundles of twisted nanofibrils (30–35 nm in diameter).^[^ ^31^ ^]^ The nanofibrils are connected with each other and upon stretching, nano‐cracks are formed rather than large cracks. This interconnection of the nanofibrils is believed to contribute to their high toughness and strength.^[^ ^32^ ^]^

The Schniepp group found that the MA silk of recluse spiders (*Loxosceles* spp.) is entirely composed of 20‐nm‐diameter fibrils with lengths over 1 µm, which exhibits a breaking strength of ≈120 nn, thus providing experimental support to the theory that nanofibrils contribute to the mechanical strength and toughness of spider silk.^[^ ^33^ ^]^ The Xia group found that nanofibrils can be formed by extruding a concentrated aqueous dope of recombinant spidroin into an acidic coagulation bath.^[^ ^34^ ^]^

#### 2.2.2 “Nano‐Fishnet” Structure

The Liu group reported that *β*‐sheet crystallites form a “nano‐fishnet”‐like structure in the amorphous region, which contributes to the remarkable mechanical properties of spider silk (Figure 2d).^[^ ^35^ ^]^ Figure 2d also shows that *β*‐crystallites are formed from the folding and packing of new *β*‐sheets on the existing ones by pleating of the repetitive protein chains.^[^ ^35^ ^]^ The *β*‐crystallites are aligned along the fiber axis during extrusion and stretching in fiber spinning, which act as nodes of the “fishnet” and enhance the fiber strength. The interconnections between intramolecular *β*‐sheets in the *β*‐crystallites highly correlate to the mechanical properties of *β*‐crystallites. The crystal size and crystallinity and their distribution in an amorphous region are different for the spider silk and the silkworm silk, thereby causing different fiber mechanical properties. For example, there are higher crystallinities in the silkworm silk (40%) than for the spider silk (22%), and the crystal size in the spider silk is finer than that in the silkworm silk. In addition, there are higher content of intramolecular *β*‐sheets compared in the spider silk (29%) than for the silkworm silk (9%), which undergoes unfolding during stretching to dissipate higher mechanical energy, causing higher toughness of the spider silk than for the silkworm silk.^[^ ^36^ ^]^ The “nano‐fishnet” structure would allow spider silk to disperse the external impact energy throughout the “fishnet”, thereby avoiding concentration of stress at a local point and preventing a crack in the structure. Consequently, the “nano‐fishnet” structure contributes to the high strength and toughness of spider silk, and provides a useful design strategy for the synthesis of high‐performance artificial fibers.

#### 2.2.3 The Multi‐Layered Core–Shell Structure

Spider dragline silks exhibit a skin–core structure, as revealed by atomic force microscopy (AFM) and electron microscopy in previous studies.^[^ ^37^ ^]^ The Weisshart group characterized the morphology and biochemical composition of the dragline silk of *Nephila clavipes*. Five layers could be clearly distinguished, consecutively arranged from the exterior to the interior in the following order: lipid layer, glycoprotein layer, skin layer, outer core, and inner core (Figure 2e).^[^ ^38^ ^]^ Only the two core layers contain known silk proteins (MaSp1 and MaSp2), which are the major contributors to the mechanical properties of spider silk.

The Weisshart group further revealed the heterogeneous distribution of MaSp1 and MaSp2 proteins at the cross‐section of the dragline silk using immunochemistry.^[^ ^18c^ ^]^ It was discovered that MaSp1 is distributed almost uniformly within the fiber's outer core, while MaSp2 is missing in the periphery but tightly packed in a certain area of the inner core with a diameter of 3–3.5 µm. The outer core enriched with the MaSp1 protein exhibits increased *β*‐sheet content, and the MaSp2 enriched in the inner core region forms an amorphous matrix because the high content and regular spacing of the proline pendant would cause less‐ordered packing of the protein macromolecular chain. Therefore, the MaSp1‐rich outer core is considered to mainly contribute to the tensile strength of the spider silk, while MaSp2 does not directly contribute to the tensile strength but exclusively to the elasticity. This heterogeneity is generated during spinning of the spidroin dope, where a laminar velocity profile exists in the flowing liquid and at a constant speed in the solid. This results in a higher flow velocity and a higher shear rate in the peripheral region compared to the core, which facilitates the formation of *β*‐sheet crystals.^[^ ^18^, ^39^ ^]^

The Numata group found that the core region of the dragline silk fiber had a predominant effect on the mechanical properties.^[^ ^40^ ^]^ The crystalline structure and mechanical properties of spider silk were studied after removing the lipid layer, glycoprotein layer, and skin layer by ether extraction, surfactant washing, and freeze–thaw cycles. The toughness, crystal structure, and supercontraction ability of the dragline silk were not significantly changed after the removal of these layers. The little influence of these skin layers on supercontraction suggests that they do not function as protection against the penetration of water molecules, but protect the fiber against protease digestion. These studies provide insights for the preparation of strong and tough fibers by mimicking the core–shell structure of natural spider silk fibers.^[^ ^37^, ^41^ ^]^

#### 2.2.4 Modification of the Mechanical Properties of the Spider Silk

Increasing the strength and toughness of a fiber is a long‐term objective in the field of fiber materials. Researchers have explored approaches to further improve the mechanical properties of spider silk. The Knez group obtained improved mechanical properties of spider silk by directly doping metals with spidroins. The metal ions were deposited into the spidroins through multiple cycles of pulsed vapor‐phase infiltration by atomic layer deposition, which could form metal coordination bonds or covalent bonds with spidroins to enhance the strength and toughness of the spider silk.^[^ ^42^ ^]^ The Steven group loaded amine‐functionalized multiwall CNTs (f‐CNTs) on natural spider silk by mechanical shearing of the aqueous dispersion of the mixture. Because of the strong polar interactions and bonding of the functional groups between the spider silk and f‐CNTs, the resulting composite fiber was ≈300% tougher than the precursor spider silk.^[^ ^43^ ^]^ In another study, the surface of spider silk was coated with a mixture of single‐walled carbon nanotubes (SWCNTs) and poly(3,4‐ethylenedioxythiophene) polystyrene sulfonate (PEDOT‐PSS), forming a composite fiber with a toughness of 420 MJ m^−3^ and a conductivity of 1077 S cm^−1^.^[^ ^44^ ^]^ Such multifunctional spider silk could be applied in the fields of bio‐electronics, sensors, wearable electronics, and artificial tendons. To increase the breaking strength of spider silk, an interesting strategy was carried out by feeding spiders with graphene or carbon nanotube (CNT) dispersion. Consequently, the spiders could produce dragline silk with enhanced mechanical properties, and its strength was comparable to or higher than that of some typical high‐strength polymeric fibers.^[^ ^45^ ^]^ This method for improving the mechanical properties of fibers could be applied to prepare other bio‐organic multifunctional materials with additional electrical, optical, and magnetic properties.

### 2.3 Artificial Fibers with High Strength and Toughness

With the in‐depth study of the structure of spider silk, great progress has been made in the preparation of strong and tough fibers using different technologies such as biotechnology, nanotechnology, polymer chemistry, and materials science. The materials used to prepare strong and tough fibers include recombinant spidroins, non‐spidroin proteins, polymer materials (e.g., hydrogels, polyurethanes, cellulose, etc.), and their composites.

#### 2.3.1 Artificial Spider Silk Based on Recombinant Spidroins

Artificially mimicking natural spider silk by employing recombinant spidroins is an effective way to prepare artificial spider silk. Recombinant spidroins are generally prepared by genetic engineering techniques, including gene design, cloning, and protein expression in different foreign hosts such as bacteria,^[^ ^46^ ^]^ yeasts,^[^ ^47^ ^]^ insect cells,^[^ ^48^ ^]^ plants,^[^ ^49^ ^]^ and mammary gland cells of goats.^[^ ^50^ ^]^ These artificial spider silk fibers are obtained from a solution of recombinant spidroins by various spinning techniques, including wet‐spinning, dry‐spinning, and electrospinning. During wet‐spinning, the solution of recombinant spidroins is extruded into a coagulation bath, where the spidroins precipitate and the fibers are formed.^[^ ^51^ ^]^ The process is widely applied to prepare recombinant spidroin fibers, and the mechanical properties of fibers are listed in **Table** **2**.

**Table 2:** The processing parameters and mechanical properties of recombinant spider silk fibers

Year	Protein	Host	Origin	Size [kDa]	Solvent	Diameter [µm]	Strength [MPa]	Strain [%]	Toughness [MJ m^−3^]	Young's modulus [GPa]	Ref.
2021	MaSp2	*E. coli*	*A. diadematus*	150	Water + Tris/HCl	27 ± 1	834 ± 34	32 ± 1	143 ± 6	5 ± 0.4	[52]
2021	MaSp1	*E. coli*	*T. clavipes*	–	Aqueous medium	34.1 ± 0.7	288.7 ± 20.9	47.1 ± 3.7	100.9 ± 13.2	3.78 ± 0.34	[34]
2020	Chimeric	*E. coli*	*E. australia/A. ventricosus*	33	Aqueous medium	27.3 ± 4	29 ± 5	20 ± 0	3.8 ± 1.2	0.5 ± 0.07	[53]
2019	AcSp1	*E. coli*	*A. trifasciata*	57	Fluorinated acid/Alcohol/Water	10 ± 1	100 ± 24	41 ± 13	37 ± 14	2.3 ± 0.7	[54]
2018	MaSp1	*E. coli*	*N. clavipes*	556	HFIP^b)^	5.7 ± 1.3	1030 ± 110	18 ± 6	114 ± 51	13.7 ± 3	[55]
2017	MaSp1	*E. coli*	*L. Hesperus/C. moluccensis*	42.7	Water + Tris/HCl	49 ± 7	282 ± 66	102 ± 24	144 ± 44	1.5 ± 0.3	[56]
2017	Chimeric	*E. coli*	*E. australia/A. ventricosus*	33	Aqueous medium	12 ± 2	162 ± 8	37 ± 5	45 ± 7	6 ± 0.8	[57]
2016	MaSp1	*E. coli*	*N. clavipes*	47	Water	8.7	286.2 ± 137.7	18.3 ± 12.8	37.7 ± 28.8	8.4 ± 4.3	[58]
2015	MaSp2	*E. coli*	*A. diadematus*	286	Water + Tris/HCl	27 ± 10	370 ± 59	110 ± 25	189 ± 33	4 ± 1	[59]
2015	MaSp1/MaSp2	Transgenic goat	*N. clavipes*	65	HFIP^b)^	29.9 ± 1.1	221.7 ± 11.0	56.0 ± 6.6	102.5 ± 13.6	6 ± 0.6	[60]
2015	MaSp1/MasSp2	Transgenic goat	*N. clavipes*	50–75	Water	–	192.2 ± 51.5	28.1 ± 26	33.8 ± 33.6	–	[61]
2014	MasSp2	*E. coli*	*A. aurantia*	86.5	HFIP^b)^	31.5 ± 4.5	53.5 ± 18.0	18.0 ± 21.6	9.3 ± 10.9	2.9 ± 1.1	[62]
2013	TuSp^a)^1	*E. coli*	*N. antipodiana*	378	HFIP^b)^	6–14	308 ± 57	10 ± 3	–	9.3 ± 3	[63]
2013	Flag	*E. coli*	*N. clavipes*	66	HFIP^b)^	15.1 ± 1.3	150.6 ± 31.3	84.5 ± 37.8	89.1 ± 23.9	–	[64]
2012	MaSp2	*E. coli*	*N. clavipes*	48	HFIP^b)^	29.1 ± 5.4	59.6 ± 19.2	4.8 ± 8.6	2.5 ± 5.4	4.3 ± 0.9	[65]
2012	Flag	*E. coli*	*N. clavipes*	58	HFIP^b)^	28.3 ± 3.4	127.5 ± 23.0	52.3 ± 23.6	54.6 ± 23.6	4.4 ± 1.0	[66]
2012	TuSp^a)^1	*E. coli*	*L. hersperus*	45	HFIP^b)^	24.5 ± 0.3	158.6 ± 7.5	13 ± 1	11.9 ± 1.6	–	[67]
2011	MaSp1	*E. coli*	*N. clavipes*	70	HFIP^b)^	17.4 ± 5	132.5 ± 49.2	22.8 ± 19.1	23.7 ± 18.5	5.7 ± 2.4	[68]
2010	MaSp1	*E. coli*	*N. clavipes*	284.9	HFIP^b)^	–	508 ± 108	15 ± 5	–	21 ± 4	[46]
2009	MaSp1	*E. coli*	*E. australia*	46	Aqueous buffers	40–90	110 ± 30	1.0 ± 0.3	–	14 ± 4	[69]
2008	MaSp2	*E. coli*	*A. aurantia*	71	HFIP^b)^	74.1 ± 33.9	49.5 ± 7.8	3.6 ± 2.6	–	0.04 ± 0.03	[70]

^a)^ (Tubuliform);

^b)^ (Hexafluoroisopropanol)

In recent years, the Scheibel group has conducted in‐depth research on the preparation of recombinant spidroin fibers.^[^ ^52^, ^56^, ^59^ ^]^ In 2015, artificial spider silk fibers with almost the same toughness as natural spider silk were obtained. The sequences mainly derived from the consensus sequences of *A. diadematus* fibroin 3 (ADF3) were used to express the recombinant spidroins in *E. coli*. The lyophilized recombinant spidroins were then dissolved and dialyzed for fiber spinning. By extruding recombinant spidroin dope into the coagulation bath followed by post‐stretching of 600%, a high tensile strength (370 ± 59 MPa), excellent extensibility (110 ± 25%), and toughness (183 ± 33 MJ m^−3^) were obtained.^[^ ^59^ ^]^ In 2007, the group designed a short major ampullate spidroin, a fusion construct between the established *L. Hesperus*‐terminal domains and the *C. moluccensis*‐core domains, which exhibited a tensile strength of 282 ± 66 MPa and toughness of 144 ± 44 MJ m^−3^.^[^ ^56^ ^]^ Natural MA silks contain at least two or more different spidroins. The Scheibel group observed possible dimerization of two different recombinant spidroins and studied their effects on the mechanical properties of spider silk. Two recombinant spidroins from *A. diadematus*, eADF3 and eADF4, were co‐produced in *E. coli*. They exhibited different physicochemical characteristics; the recombinant variant eADF3 assemblies showed a micellar shape, and the recombinant eADF4 assembled into nanosized cross‐*β*‐fibrils. The homo‐ and heterodimers of eADF3 and eADF4 are formed by CTD, and heterodimers self‐assemble into fibrillary networks. The artificial spider silk obtained from the heterodimers exhibited a tensile strength of 834 ± 34 MPa, extensibility of 32 ± 1%, and toughness of 143 ± 6 MJ m^−3^.^[^ ^52^ ^]^

The molecular weight of the recombinant spidroins also affects the mechanical properties of the artificial spider silk. The Lee group found that the fibers showed enhanced mechanical properties from high‐molecular‐weight spidroins, and they obtained a tensile strength of 508 MPa, extensibility of 15%, and Young's modulus of 21 GPa from a 284.9 kDa recombinant spidroin.^[^ ^46^ ^]^ The Zhang group developed a synthetic biology approach to produce high molecular weight recombinant spidroins (556 kDa) that contain 192 repeating motifs of the *N. clavipes* dragline spidroin, which showed a tensile strength of 1.03 ± 0.11 GPa, an extensibility of 18 ± 6%, a toughness of 114 ± 51MJ m^−3^, and a Young's modulus of 13.7 ± 3.0 GPa.^[^ ^55^ ^]^

In addition, the mechanical properties of the artificial spider silk are also correlated to the concentration of the spinning dope and coagulation bath. For example, it is generally difficult to achieve natural spidroins’ concentration (30–50%, w/v) with recombinant spidroins.^[^ ^17a^ ^]^ The Johansson group designed a minispidroin composed of a highly soluble NTD from *Euprosthenops australis* MaSp1 and a very soluble CTD from *A. ventricosus* MiSp, bracketing a short repetitive region from *E. australis*. The dope concentration reached 50%, and the obtained fiber exhibited a tensile strength of 162 ± 8 MPa and a toughness of 45 ± 7 MJ m^−3^.^[^ ^57^ ^]^

**Figure 3:**
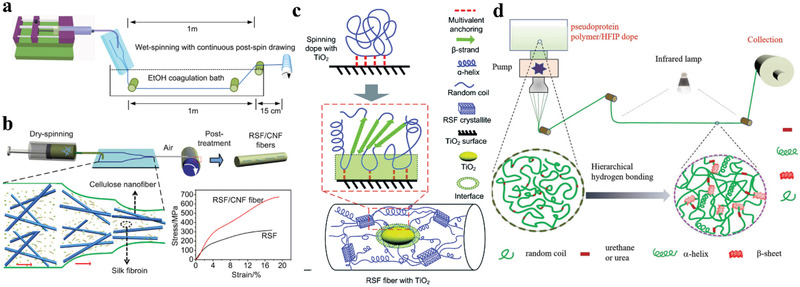
a) Schematic microfluidic spinning of the recombinant spider silk fiber. Reproduced under the terms of the Creative Commons CC BY license.^[^ ^58^ ^]^ Copyright 2016, Springer Nature. b) Schematic of the RSF/CNF fibers by dry‐spinning using a microfluidic channel. Reproduced with permission.^[^ ^75a^ ^]^ Copyright 2019, American Chemical Society. c) Proposed model of the nano‐confined crystallite toughening mechanism of hybrid artificial silks. Reproduced with permission.^[^ ^75b^ ^]^ Copyright 2014, Royal Society of Chemistry. d) Fabrication of the pseudoprotein polymer fiber. Reproduced with permission.^[^ ^81^ ^]^ Copyright 2019, Wiley‐VCH.

The Xia group designed a microfluidic spinning chip to mimic the natural spinning apparatus, including shearing and elongational sections. (**Figure** **3**a) The spinning dope of recombinant spidroins flowed through the microfluidic spinning chip, causing self‐assembly and orientation of spidroins to form fibril structures. The obtained artificial fiber exhibited a tensile strength of 510 MPa and an extensibility of 15%.^[^ ^58^ ^]^ This research group further designed a spidroin‐mimic composed of CTD and NTD bracketing 16 consensus repeats of the core region from *T. clavipes*. The obtained fiber exhibited a tensile strength of 288.7 ± 20.9 MPa, an extensibility of 47.1 ± 3.7%, a toughness of 100.9 ± 13.2 MJ m^−3^, and a Young's modulus of 3.78 ± 0.34 GPa. Nanofibrils were not observed in the concentrated aqueous dope of recombinant spidroins lacking CTD or NTD, and decreased mechanical properties were also observed.^[^ ^34^ ^]^ The synergy of the terminal domains of recombinant spidroins for fibril formation and microfluidic wet‐spinning for controlling and regulating the spinning process contributed to the extraordinary mechanical properties of the artificial spider silk. Apart from the increased alignment, post‐stretching also led to an increase in *β*‐sheet content.^[^ ^68^ ^]^

#### 2.3.2 Artificial Strong and Tough Fibers From Non‐Spidroin Proteins and Polypeptides

The microstructure of silk fibroin from silkworm silk is similar to that of spidroin, including antiparallel *β*‐sheet nanocrystals and an amorphous matrix consisting of *α*‐helices and random coils.^[^ ^30^, ^71^ ^]^ Therefore, regenerated silk fibroin (RSF) is one of the most important resources for preparing artificial strong and tough fibers. The Zhang group conducted abundant research on the preparation of RSF fibers.^[^ ^72^ ^]^ The RSF solution was obtained by dissolving degummed silk in a LiBr aqueous solution, followed by dialysis. The RSF fibers were then prepared using capillary spinning equipment. The tensile strength of the fiber obtained by this method was 46 MPa, which could be further increased to 359 MPa after a preliminary post‐stretch in an ethanol aqueous solution.^[^ ^72a^ ^]^ The orientation of the crystalline and molecular chains, crystallinity, and *β*‐sheet content increased after post‐stretching.^[^ ^72b^ ^]^ To further obtain an ultra‐high‐performance biomimetic fibers, the Zhang group prepared RSF fibers from RSF solution using a biomimetic microfluidic chip. The resulting artificial spider silk exhibited a tensile strength of 614 MPa and an extensibility of 27%.^[^ ^72c^ ^]^ The Scheibel group prepared collagen fibers using a microfluidic chip, which exhibited a tensile strength of 383 ± 85 MPa and a Young's modulus of 4.14 ± 0.5 GPa,^[^ ^73^ ^]^ exceeding those of the collagen fibers produced by wet‐spinning.^[^ ^74^ ^]^ The Zhang group further composited RSF with nanomaterials to spin high‐performance fibers.^[^ ^75^ ^]^ Cellulose nanofibers (CNFs) were introduced into the RSF solution, and increased crystallinity and mechanical properties were observed by the formation of a CNF network and its interaction with RSF.^[^ ^75^, ^76^ ^]^ (Figure 3b) An RSF/TiO_2_ composite fiber exhibited a strength of 218.5 MPa, an extensibility of 88.3%, and a toughness of 93.1 ± 27.1 MJ m^−3^. Nanomineral TiO_2_ exhibits a large specific surface area and rich titanium and oxygen atoms, which can form hydrogen bonds (H‐bonds) and coordination complexes with RSF to form nanoconfined crystallites and inhibit the conformational transition of random coil/*α*‐helix to *β*‐sheet (Figure 3c).^[^ ^75b^ ^]^ The principle can be extended to the dry‐spinning of RSF and graphene oxide (GO), which exhibit a tensile strength of 435.5 ± 71.6 MPa and extensibility of 35.9%.^[^ ^76c,d^ ^]^ Such a concept‐confined network provides a new approach for the preparation of high‐performance artificial fibers.

The synergistic effect of the rigid *β*‐crystallites embedded in the soft amorphous regions to form crosslinks is an important structural model for obtaining high tensile strength and toughness of the spider silk, and it can be extended to non‐spidroin proteins. The Liu group fabricated strong and tough fibers from globular bovine serum albumin (BSA) protein by employing microfluidic spinning, which was achieved by pumping a high‐concentration bovine serum albumin (BSA) solution and the glutaraldehyde (GA) solution into the outer and inner channels, respectively, of a double‐channel glass capillary microfluidic device with a tapered outlet, followed by collection by a rotatory collector. The mechanical strength of the obtained fiber was further enhanced by the crosslinking reaction of aldehyde and amino groups, as well as by the alignment of the protein chains induced by post‐stretching, reaching 300 MPa.^[^ ^77^ ^]^ In another study by the Liu group, a chimeric protein, consisting of the sequences of a cationic elastin‐like polypeptide (ELP) and a squid ring teeth (SRT) protein, was crosslinked with GA and a fiber was obtained by microfluidic spinning. The pre‐crosslinking of ELP lysine residue with GA was performed in a mixture solution of the SRT–ELP protein and GA in a syringe, followed by further crosslinking with GA in a coagulation bath. The obtained fiber after post‐stretching exhibited a breaking strength of 630 MPa and toughness of 130 MJ m^−3^.^[^ ^78^ ^]^

Since proteins are made of polypeptide chains, mimicking the structure of proteins using polypeptides is an efficient way to prepare artificial spider silk. A multiblock polypeptide was synthesized to mimic the secondary structures of spider silk using a two‐step chemical synthesis method.^[^ ^79^ ^]^ Chemoenzymatic polymerization was employed in the first step to synthesize two types of polypeptide fragments. One was polyalanine, capable of forming *β*‐sheets that mimic the crystalline region of the spider silk; the other was poly(glycine‐random‐leucine) that mimicked the soft amorphous region. The second step was to ligate the two polypeptide fragments by post‐polycondensation using polyphosphoric acid as the condensing agent. The resulting multiblock polypeptides formed an antiparallel *β*‐sheet crystalline region and an amorphous region, which mimicked the structure of the natural spider silk.^[^ ^79^ ^]^ This work provides a promising method for the chemical synthesis of artificial spider silk by mimicking the secondary structure of spider silk. Further work is required to increase the molecular weight of the synthesized polypeptide so that the mechanical properties can be further improved.

The Floudas group found that the amine‐terminated polypeptide poly(*γ*‐benzyl‐1‐glutamate) (PBLA) formed an *α*‐helix if the degree of polymerization was higher than 18, and both *α*‐helices and *β*‐sheets existed if the degree of polymerization of PBLA was less than 18.^[^ ^80^ ^]^ By controlling the polymerization degree of PBLA at ≈15, the Hu group prepared a PBLA that exhibited a unique composition of *α*‐helices and *β*‐sheets.^[^ ^81^ ^]^ They further chemically linked the PBLA with random‐coil‐like polymer chains (poly(tetramethylene ether glycol)) through urea linkage to prepare a pseudoprotein by mimicking the fibroin. Then, the fiber was prepared by dry‐spinning (Figure 3d). Such pseudoprotein polymer fibers simulated the natural spinning process of spider silk, which exhibited a tensile strength of 100 MPa and a toughness of 387 MJ m^−3^. The preparation method of this pseudoprotein polymer fiber is simple, and the raw materials are widely available, which facilitates large‐scale manufacturing.^[^ ^81^ ^]^ The Qiao group mimicked the structure of the crystalline region and the amorphous region of spider silk by using a hydrogel network containing polypeptides. An amorphous hydrogel network containing primary amine group pendants was prepared by free radical polymerization. Poly(valine) and poly(valine‐*γ*‐glycine) were then grafted onto the hydrogel network by amine‐initiated N‐carboxyanhydride ring‐opening polymerization (NCA‐ROP). This served as *β*‐sheet nanocrystals. The resulting fibers exhibited reinforced compressive strength and stiffness after the incorporation of poly(valine) and poly(valine‐*γ*‐glycine).^[^ ^82^ ^]^ The N‐carboxyanhydride ring‐opening polymerization (NCA‐ROP) is a cheap and simple method for in situ grafting of long‐chain polypeptides. It provides the possibility for large‐scale production of polypeptide‐based materials with high strength and toughness.

#### 2.3.3 Artificial Strong and Tough Fibers by Polymer Materials

**Figure 4:**
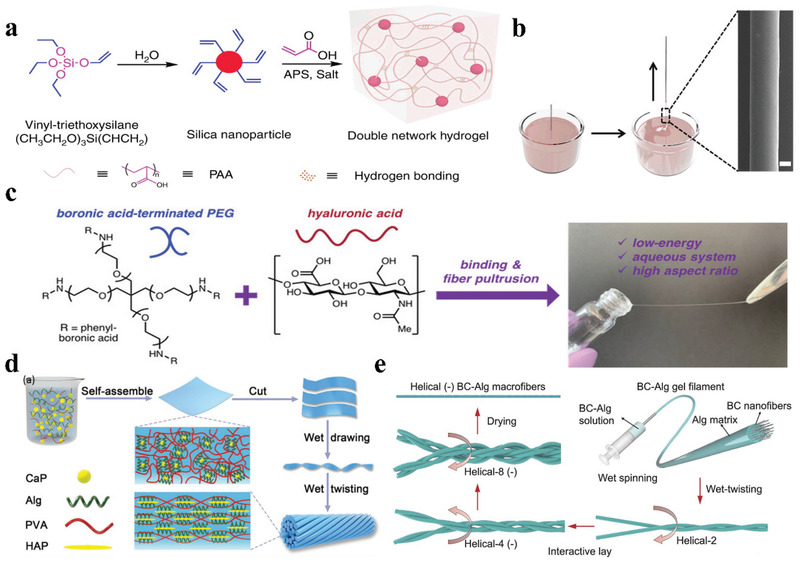
a) Synthesis of polyacrylic acid hydrogel by free radical polymerization employing acrylic acid as the monomer, ammonium persulfate (APS) as the initiator, and vinyl‐functionalized silica nanoparticles as the cross‐linker. b) A single fiber being draw‐spun by dipping a steel rod into the hydrogel reservoir. The hydrogel fiber shown in the micrograph contained 0.1 wt% VSNPs. Scale bar: 10 µm. Reproduced under the terms of the Creative Commons CC BY license.^[^ ^83^ ^]^ Copyright 2019, Springer Nature. c) A polymer fiber being draw‐spun from a bulk hydrogel precursor composed of bionic acid‐terminated PEGs and HA, which formed non‐covalent dynamic interactions. Reproduced with permission.^[^ ^84^ ^]^ Copyright 2020, American Chemical Society. d) Schematic illustration of the preparation process and network microstructure of the PVA/Alg/HAP hybrid macrofiber. Reproduced with permission.^[^ ^88^ ^]^ Copyright 2019, Wiley‐VCH. e) Schematic illustration of the fabrication process of the bioinspired hierarchical helical BC–Alg macrofibers. At each structural level, every two sub‐level gel filaments are twisted together to prepare a higher‐level helical fiber. Helical‐2, 4, and 8 indicate helical fibers composed of 2, 4, and 8 original filaments, respectively. The helical fibers with the same twist direction (Albert lay) at each level are defined as helical (+). The helical fibers with opposite twist directions (interactive lay) at each level are defined as helical (−). Reproduced under the terms of the Creative Commons CC BY license.^[^ ^93^ ^]^ Copyright 2019, Oxford University Press.

Hydrogels are hydrophilic 3D networks through physical or chemical crosslinking in water, which can be shaped into various forms such as bulks, films, and fibers, for different applications. Hydrogel fibers are important for the artificial preparation of strong and tough fibers. Based on the core–shell structure of the natural spider silk, the Liu group prepared polyacrylic acid (PAA) hydrogel fibers crosslinked by vinyl‐functionalized silica nanoparticles, which formed a hierarchical core–shell structure during water‐evaporation‐controlled self‐assembly of the polymer chains.^[^ ^83^ ^]^ The hydrogel fibers can be drawn from a bulk hydrogel by dipping a thin metal wire in the hydrogel, and the fiber diameter can be controlled precisely by the dipping depth (**Figure** **4**a,b,). The mechanical properties of these artificial spider silk fibers could be further reinforced by ion doping and twist insertion, which exhibited a tensile strength of 895 MPa, a high toughness of 370 MJ m^−3^, and a damping capacity of 95%. Such a high energy dissipation ability could have potential applications in kinetic energy buffers and shock absorption. The Anderson group obtained hydrogel fibers from bulk hydrogels with physical crosslinks using the draw‐spinning method.^[^ ^84^ ^]^ As shown in Figure 4c, the hydrogel fibers were prepared by drawing a thread from the bulk gel precursor composed of boronic‐acid‐functionalized branched 4‐arm polyethylene glycol polymer (PEG) and hyaluronic acid (HA), where dynamic physical crosslinks were formed in the hydrogel. The elastic modulus, tensile strength, and strain of the polymer fibers could be customized by designing and tuning the polymer structure and concentration. The diameter of the polymer fibers ranged between 4–20 µm, and the maximum fiber length reached 10 m. Artificial spider silk fiber with adjustable mechanical properties can be employed in multiple environmental applications for energy absorption and dissipation, impact protection, and implantable biomedical applications.

Spinning hydrogel fibers is not easy because of the 3D network structure. Recently, there has been some progress in this regard. The Zhu group conducted in‐depth research on the preparation of hydrogel fibers by employing advanced spinning strategies.^[^ ^85^ ^]^ The dynamic‐crosslinking‐spinning (DCS) is an effective way to continuously prepare hydrogel fibers. The poly(ethylene glycol) diacrylate (PEGDA) solution was squeezed into the water bath through a syringe needle to form a fiber, which was further polymerized to form a stable 3D network by UV irradiation. The resulting fibers rapidly absorbed water owing to the large specific surface area, and the diameter of the hydrogel fiber was controllable.^[^ ^86b,d^ ^]^ The incorporation of cellulose nanocrystals (CNCs) into the PEGDA solution enhanced the mechanical properties of the hydrogel fibers spun by the DCS method.^[^ ^85e^ ^]^ Such a dynamic wet‐spinning strategy could provide the possibility to produce hydrogel fibers with a wide variety of properties for potential applications in flexible sensing, wearable electronics, and biomedical applications.^[^ ^86^ ^]^ The Wang group prepared stretchable, conductive, and self‐healing hydrogel fibers by dry‐wet spinning. The poly(N‐acryloylglycinamide‐co‐acrylamide) (PNA) hydrogel precursor was heated to ≈80 °C in a storage tank. The precursor softened and transformed from a gel state to a viscous sol state. The PNA hydrogel fiber was obtained by extruding the softened PNA hydrogel out of the spinning head, and then solidified into a gel fiber in an ethyl acetate coagulation bath at room temperature. The fiber so achieved had a tensile strength of 2.27 MPa, a stretchability of 900%, and a conductivity 0.69 S m^−1^.^[^ ^87^ ^]^ The surface of the PNA hydrogel fibers was coated with elastomeric poly(methyl acrylate) (PMA) so that the PNA/PMA core–sheath fiber could resist water evaporation and absorption.

The Tang group prepared strong and tough fibers from hierarchically‐structured organic–inorganic composites by imitating the rigid crystalline and the flexible amorphous protein blocks of spidroins. As shown in Figure 4d, a composite film containing sodium alginate (Alg), calcium phosphate (CaP), hydroxyapatite (HAP), and polyvinyl alcohol (PVA) was prepared, and the fibers were obtained by twisting the strips that were cut from the film. The obtained fiber exhibited a tensile strength of 950 MPa, toughness of 296 J g^−1^, and breaking strain of 80%, which were comparable to those of natural spider silk.^[^ ^88^ ^]^ The Ma group prepared a multifunctional composite fiber with a core–shell structure by coating a thin layer of PMA on sodium polyacrylate hydrogel (PAH) fibers. The obtained fiber contained reversible transformations of crystalline and amorphous domains, which exhibited a tensile strength of 5.6 MPa, breaking strain of 1200%, fast resilience of <30 s, electrical conductivity of 2 S m^−1^, and good anti‐freezing properties (retained stretchability and conductivity at −35 °C).^[^ ^89^ ^]^

In addition to hydrogels, polyurethane fibers are employed to mimic spider silk to obtain high strength and toughness. The *β*‐sheet nanocrystals in spider silk were homogeneously embedded in the amorphous matrix, which consisted of hydrogen‐bonded (H‐bonded) polypeptide chains. The excellent mechanical robustness of the spider silk mainly originated from the unique H‐bond arrays in the *β*‐sheet nanocrystals.^[^ ^29^, ^32^, ^90^ ^]^ Inspired by the spider silk, the Liu group prepared supramolecular elastomers by designing abundant H‐bonding segments consisting of multiple acylsemicarbazide and urethane moieties linked with flexible alicyclic hexatomic spacers. The H‐bond arrays could dissipate energy more efficiently by acting as firm but reversible crosslinks and sacrificial bonds. The elastomer achieved a breaking stress of 75.6 MPa and a toughness of 390.2 MJ m^−3^.^[^ ^91^ ^]^

Bacterial cellulose (BC) nanofibers produced by bacterial fermentation exhibit a high degree of polymerization and crystallinity, making them suitable building blocks for the fabrication of high‐performance fibers.^[^ ^92^ ^]^ The Yu group prepared BC nanofiber‐based hierarchical nanocomposite fibers by combining a facile wet‐spinning process with a subsequent multiple wet‐twisting procedure (Figure 4e). The twisted filaments entangled together via surface adhesion upon drying, resulting in dense macrofibers with decreased voids, and exhibited a tensile strength of 535.4 MPa, breaking strain of 16.2%, and toughness of 44.8 MJ m^−3^.^[^ ^93^ ^]^ The Söderberg group conducted in‐depth research on the fabrication of strong fibers using cellulose nanofibrils obtained from trees.^[^ ^94^ ^]^ Homogeneous and smooth fibers were prepared from a low‐concentration dispersion of cellulose nanofibrils in water by combining a surface‐charge‐controlled gel transition with a hydrodynamically‐induced fibril alignment. The cellulose nanofibrils in the liquid dispersion can rotate freely owing to strong electrostatic repulsion. The flow of the electrolyte solution drove the nanofibrils to rearrange along the flow direction. Finally, with the diffusion of the electrolyte, the gel fiber with an aligned structure was formed stably by reducing the electrostatic repulsion between the nanofibrils. The resulting fiber showed a tensile strength of 490 ± 86 MPa and an extensibility of 6.4 ± 1.6%.^[^ ^94a^ ^]^ According to this principle, the Söderberg group further fabricated super‐strong cellulose fibers using an improved double‐flow‐focusing channel device. The increased sheath flow of water supported electrostatic repulsion and prevented the core flow from contacting the channel walls, resulting in a tensile strength of 1570 MPa and a Young's modulus of 86 GPa.^[^ ^94c^ ^]^

## 3 Other Physical Properties of the Spider Silk and the Inspired Artificial Fibers

In addition to its excellent mechanical properties, spider silk exhibits many other interesting physical properties, including humidity/water response, water collection, light transmission, torsional energy dissipation, shape‐memory, and thermal conductance. This has inspired researchers to biomimetically prepared artificial fibers. Here, we reviewed the physical properties of spider silk and the artificial functional fibers thus inspired.

### 3.1 Humidity/Water Response

#### 3.1.1 Humidity/Water Response of Spider Silk

Spider silk is a natural biological material that is highly sensitive to humidity and water because of hydrophilic peptide segments in spidroins and surface coatings.^[^ ^95^ ^]^ The mechanical properties of dragline silk are optimal in a suitable ambient environment, which changes drastically if the fibers are wet or in a high‐moisture environment.^[^ ^96^ ^]^ Studies have found that water can disrupt the H‐bonds to increase the molecular mobility, so that the molecular entropy could drive the non‐crystalline regions to rearrange to lower energetic configurations.^[^ ^13^, ^97^ ^]^ When the relative humidity of spider silk exceeded 60%, the diameter of the spider dragline silk increased and the fiber length decreased by more than 50%, which is commonly referred to as supercontraction behavior.^[^ ^4^, ^22^, ^97^ ^]^ This supercontraction could tighten the spider web in rain or dew to ensure the integrity and shape of the web. The stress generated by supercontraction in restrained dragline silk can reach 40–50 MPa when the relative humidity is over 70%, and the generated stress can exceed 100 MPa in rapid supercontraction. This quality can be harnessed to prepare artificial muscles, sensors, and soft robots.^[^ ^98^ ^]^

**Figure 5:**
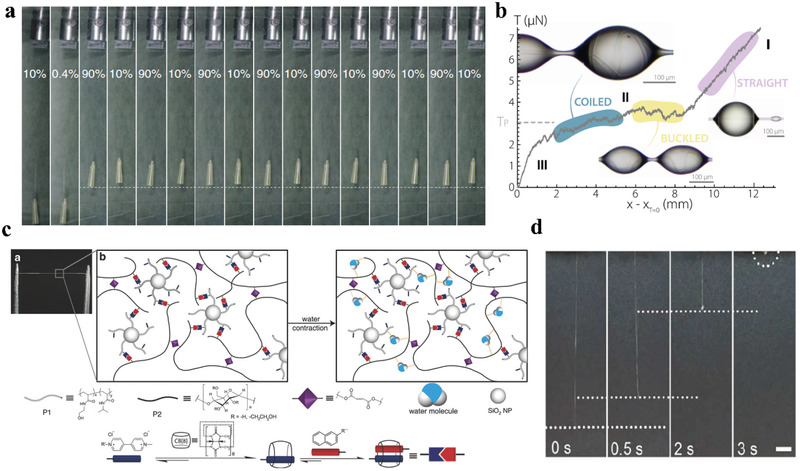
a) Lifting of an object by spider dragline silk during moisture‐driven supercontraction and cyclical lifting of the objects by periodically changing the humidity. Reproduced with permission.^[^ ^99^ ^]^ Copyright 2009, Company of Biologists Ltd. b) Force–displacement relationship during coiling of a spider capture silk in water droplets by stress relaxation, which showed a behavior of solid–liquid transition. Reproduced with permission.^[^ ^100^ ^]^ Copyright 2016, National Academy of Sciences (United States). c) Schematic illustration of the hydrogel consisting of P1 grafted onto the silica nanoparticles, P2, cucurbit[8]uril, which exhibited a double‐crosslinked network, including the physical interactions (between P1 and P2) and covalent crosslinks in P2 (formed by an additional UV‐crosslinking step). Reproduced under the terms of the Creative Commons CC BY License.^[^ ^106^ ^]^ Copyright 2018, Wiley‐VCH. d) Snapshots of a 5‐cm‐long hydrogel fiber supercontracted into a 50‐µm‐diameter ball at 95% RH. Scale bar: 1 cm. The white dashed lines indicate the supercontraction process of hydrogel fibers, and the white dashed oval presents the hydrogel small ball. Reproduced under the terms of the Creative Commons CC BY license.^[^ ^83^ ^]^ Copyright 2019, Springer Nature.

Spider dragline silk can contract to different extents in response to environmental humidity, which makes it possible to simulate the performance of biological muscles. The Blackledge group found that spider silk could contract and elongate reversibly by alternatively changing the relative humidity between low and high values.^[^ ^99^ ^]^ As shown in **Figure** **5**a, a 9.5 mg load was suspended at the end of a 5.5‐µm‐diameter spider silk, which was subjected to cyclic changes in the relative humidity. As the relative humidity first increased from 0.4% to 90%, supercontraction of the spider silk occurred, and a large, irreversible contractile stroke was generated. Subsequently, the spider silk showed a reversible change in length by 1.7% of the total post‐supercontracted length when the relative humidity was cyclically changed between 10% and 90%. This repeated ascension process produced a work capacity 50 times that of a human muscle.

The spider capture silk is covered with glue‐like droplets to adhere to the prey, which also results in the hydrophilicity of the capture silk. The Antkowiak group found that the capture silk had a novel solid–liquid behavior when it was soaked in a water droplet.^[^ ^100^ ^]^ As shown in Figure 5b, the capture silk tended to shrink in a water droplet to form a coil and generate stress, which exhibited a solid‐to‐liquid transition upon stress relaxation. During the contraction, it was similar to a conventional elastic solid in region I, where the tension decreased almost linearly with the decrease in displacement. In region II, the capture silk changed from solid‐like to liquid‐like and began to bend in the droplets, where the tension was almost unaffected by the displacement. In region III, the capture silk was crimped in the droplets and accumulated until the overall tension was relaxed, where the length of the spider capture silk could shrink without limit and behave like a liquid. Based on the above phenomena, the Zhu group found that water droplets could trigger the contractile actuation of spider silk by forming a coil, which could be employed to lift heavy objects.^[^ ^101^ ^]^ A certain weight was hung at the bottom end of the spider silk, and water droplets were slowly added from the top of the silk fiber. As the volume of the water droplets gradually increased, the spider silk continued to contract, forming a coil and lifting the weight to a certain height. As the water droplets became larger, they fell out and the coiled spider silk unfolded for the next cycle for lifting. This load‐lifting process is energy‐saving and environment‐friendly, and can be applied to bionic muscles for soft robotics or load lifting equipment.

Spider silk not only exhibits contractile actuation driven by moisture and water, but also shows torsional rotation in a humid atmosphere. The Buehler group accurately controlled the torsional actuation of a spider dragline silk by adjusting the relative humidity. The silk underwent a torsional rotation of 300° mm^−1^ at a relative humidity of 70%.^[^ ^102^ ^]^ This humidity‐induced torsional rotation of spider dragline silk is related to its supercontraction behavior. The molecular simulation of the MaSp1 and MaSp2 proteins in the dragline silk illustrated that the proline in MaSp2 should be the origin of this unique torsional behavior, where the H‐bonds in the proline rings are destroyed in an asymmetric manner after interaction with water molecules, resulting in unidirectional torsional rotation of the spider dragline silk. This humidity‐induced torsion of spider dragline silk can be applied to next‐generation biosensors, artificial muscles, and hygrometers.

The sensitive humidity response can be employed as a photonic component for humidity sensing, for possible applications in environmental monitoring, food detection, bioengineering, information, and communications.^[^ ^103^ ^]^ The Yuan group employed spider egg sac silk (SESS) from *A. ventriculosus* as a humidity‐sensitive photonic sensor.^[^ ^104^ ^]^ The surface of the SESS was in vertical contact with the tapered fiber, and the diameter of the SESS increased with an increase in the ambient humidity, causing the whispering‐gallery‐mode resonance dips to shift. The humidity sensor exhibited an average sensitivity of 389.1 pm per 1% relative humidity change for the investigated humidity range of 20% to 75%. The same research group configured another humidity sensor by employing a single‐mode fiber (SMF) and SESS.^[^ ^105^ ^]^ When the ambient humidity increased, the length of the interference cavity decreased due to increased diameter of the spider silk, which led to a red shift of the interference spectrum. The maximum sensitivity of the humidity sensor was 0.99 nm per 1% relative humidity change. Because of its biocompatibility and high sensitivity, spider silk has broad applications in biochemical and bio‐optical sensing.

#### 3.1.2 Artificial Fibers with Humidity/Water Responsive Supercontraction

Humidity‐responsive supercontraction was also realized upon mimicking spider silk. The Scherman group prepared humidity‐sensitive supramolecular fibers by draw‐spinning of a composite hydrogel consisting of methyl viologen‐functionalized polymer (P1) grafted onto silica nanoparticles, naphthol‐functionalized hydroxyethyl cellulose (P2), cucurbit[8]uril, and a photoinitiator (Irgacure 2959). As shown in Figure 5c, the cucurbit[8]uril‐crosslinked P1 and P2 formed a dynamic heteroternary complex with pendant guest molecules on both polymers in aqueous solution. The resulting fiber exhibited a double‐crosslinked network, including physical interactions between P1 and P2, and covalent crosslinks in P2 (formed by an additional UV‐crosslinking step). At high humidity, the H‐bonds between the polymer chains are disrupted when the water molecules penetrate the fiber matrix. This did not cause any collapse or breakage of the double‐crosslinked fiber, but enabled the polymer chains to reconfigure, and the fiber contracted up to 50% of its original length.^[^ ^106^ ^]^ In addition, the hydrogel fiber prepared by the Anderson group can supercontract by 75% in response to high humidity,^[^ ^84^ ^]^ and the maximum supercontraction degree of PAA hydrogel fiber prepared by our group can reach nearly 100% (Figure 5d).^[^ ^83^ ^]^

Inspired by the crystalline structure of spider silk, a polyurethane‐nanocomposite fiber was prepared by wet‐spinning. The spinning dope solution was obtained by mixing hydrophilic polyurethane as the matrix and nanocarbons (e.g., GO and CNT) as the toughening additives. The mixture was injected into a water coagulation bath to form the fibers. Upon water absorption, the stretched polymer chains could be rearranged, resulting in a fiber supercontraction of 59%.^[^ ^107^ ^]^

### 3.2 Water Collection

#### 3.2.1 Water Collection of the Spider Silk

**Figure 6:**
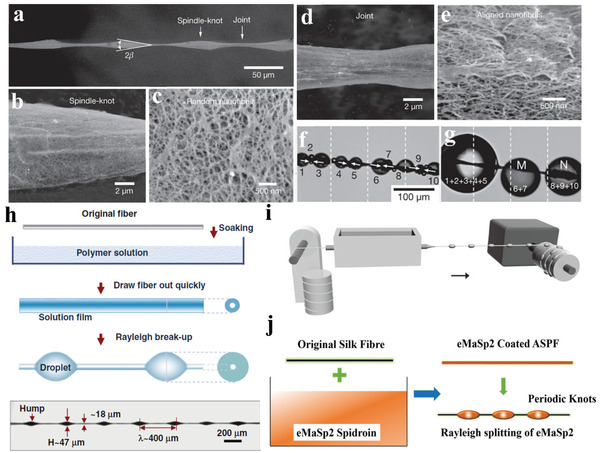
a) Environmental SEM images of the periodic spindle‐knots linking with slender joints of the spider silk. The apex angle of the spindle‐knots (2*β*) is about 19°. b) Low‐ and c) high‐magnification images showing that the spindle‐knot is randomly interweaved by nanofibrils. d) Low‐ and e) high‐magnification images of the joint, which is composed of axially aligned nanofibrils. f) Small water droplets condensing on the spider silk (denoted 1–10). The water droplets move directionally from the joints to the spindle‐knots (as indicated by arrows) and increase in volume. g) The smaller water droplets (1–5) coalesce to a larger water droplet, while the water droplets (6, 7, and 8–10) coalesce to two medium water droplets. Reproduced with permission.^[^ ^108^ ^]^ Copyright 2010, Springer Nature. h) Scheme of fabricating artificial fibers with spindle‐knot structure. Reproduced with permission.^[^ ^110^ ^]^ Copyright 2011, Wiley‐VCH. i) Schematic illustration of the fluid coating method used for large‐scale fabrication of artificial fibers with spindle‐knot structure. Reproduced with permission.^[^ ^112^ ^]^ Copyright 2011, Wiley‐VCH. j) An all silk‐protein fiber consisting of *B. mori* degummed silk coated with recombinant MaSp2. The spindle‐knot structure was generated by coating a thin layer of MaSp2 by drawing the fiber out of the dope, which subsequently split up into knots with periodic intervals. Reproduced with permission.^[^ ^116^ ^]^ Copyright 2020, Wiley‐VCH.

Spider silk exhibits a water collection capability from air because of its unique fiber morphology and chemical composition.^[^ ^51^ ^]^ The Jiang group reported the directional flow of water droplets on the capture silk of the cribellate spider *Uloborus walckenaerius*, which showed a spindle‐knot structure contributing to the function of water collection (**Figure** **6**a).^[^ ^108^ ^]^ Low‐ and high‐magnification images revealed that the spindle‐knot was composed of random nanofibrils (Figure 6b,d), and the joint was covered with aligned nanofibrils parallel to the axial direction of the spider silk (Figure 6c,e). This curvature gradient between the spindle knots and the joints generates a Laplace pressure difference and surface energy gradient, which is highly correlated to the chemical composition and surface roughness.^[^ ^109^ ^]^ The synergistic effect of the surface energy gradient and the Laplace pressure difference enables the small droplets on spider silk to merge into a large droplet and transmit from the joints to the spindle knots (Figure 6f,g).

#### 3.2.2 Artificial Fibers with Water Collection Capacity

Artificial fibers with periodic spindle knots and joints were designed to collect water from foggy or humid atmospheres. The Jiang group prepared these fibers by soaking nylon fiber in a poly(methyl methacrylate) (PMMA) solution in *N*,*N*‐dimethyl formamide (DMF). The fiber was then drawn out at a speed of 3 mm s^−1^. After evaporating the DMF solvent, droplets were formed intermittently along the fiber because of Rayleigh instability (Figure 6h). The threshold volume, which was used to evaluate the adhesive ability to drops of the fiber, refers to the volume of a drop just before it detached from a given fiber. The artificial fiber exhibited a threshold volume of 4.38 ± 0.13 µL, which exceeded twice that (2.03 ± 0.11 µL) of the uniform fibers, indicating stronger adhesive ability of the artificial fibers.^[^ ^110^ ^]^ The Jiang group also prepared artificial fibers with different spindle knot sizes by regulating the solution concentration and fiber‐drawing speed, where the fiber with an optimized average spindle‐knot size of 0.12 nL could collect highest amount of water (34 nL).^[^ ^111^ ^]^ These results suggest a feasible method for preparing artificial fibers with high water collection capacity.

Jiang et al. further designed a fluid‐coating apparatus to prepare fibers with intermittent spindle knots on a large scale in an inexpensive manner (Figure 6i). The nylon fiber was fixed horizontally in the polymer solution reservoir through the center of the capillary tube with one end connected to the motor. When the fiber was continuously stretched out through the solution reservoir, it was uniformly coated with a solution film at the end of the capillary tube. The cylindrical polymer solution film breaks into dispersed droplets along the fiber due to Rayleigh instability and finally changes to spindle knots after solvent evaporation.^[^ ^112^ ^]^ In addition to dip‐coating and fluid‐coating methods, there are other effective ways to fabricate bio‐inspired fibers for collecting water from fog, such as electrodynamic^[^ ^113^ ^]^ and microfluidic^[^ ^114^ ^]^ methods. The nylon fiber in the above‐mentioned process can also be replaced with other types of fibers, such as glass fibers and copper wires, for different applications.^[^ ^115^ ^]^ In 2020, the Hu group prepared an all‐silk‐protein fiber consisting of *Bombyx mori* degummed silk coated with recombinant MaSp2,^[^ ^116^ ^]^ (Figure 6i), which exhibited dispersed spindle knots and joints and showed a high water collection capacity.^[^ ^112^ ^]^

Spider‐silk‐inspired water collection is a green way to obtain water without an external energy supply. In addition, by employing different types of polymer materials with diverse electrical, optical, and magnetic properties, broad application prospects can be integrated with controllable liquid flow for analysis, sensing, chemical reaction, and oil–water separation.

### 3.3 Light Transmission

#### 3.3.1 Light Transmission of the Spider Silk

An optical fiber is used for transmitting data along a long fiber that is usually made of plastic or glass. Spider silk shows high transmittance and glistens in sunlight, which motivated researchers to study its optical transmission capacity. In 2003, the Bêche group demonstrated that natural spider silk can be employed as an eco‐friendly and efficient optical fiber.^[^ ^117^ ^]^ A straight spider silk fiber exhibited an attenuation coefficient of 10.5 dB cm^−1^ in air. Successful light propagation was achieved for spider silk in a loop shape, spider silk immersed in physiological liquid, and for silk fiber integrated into photonic chips by hybridizing with synthetic polymers (photoresist). No destruction of the spider silk was observed during UV‐lithography for integration in the photonic chips, and efficient micro‐optical coupling between the silk and synthetic optical components was observed. These optical performances combined with unique biocompatibility, bioresorbability, flexibility, high strength, and robustness of the spider silk are very promising for biophotonics applications.

#### 3.3.2 Artificial Fibers for Light Transmission

**Figure 7:**
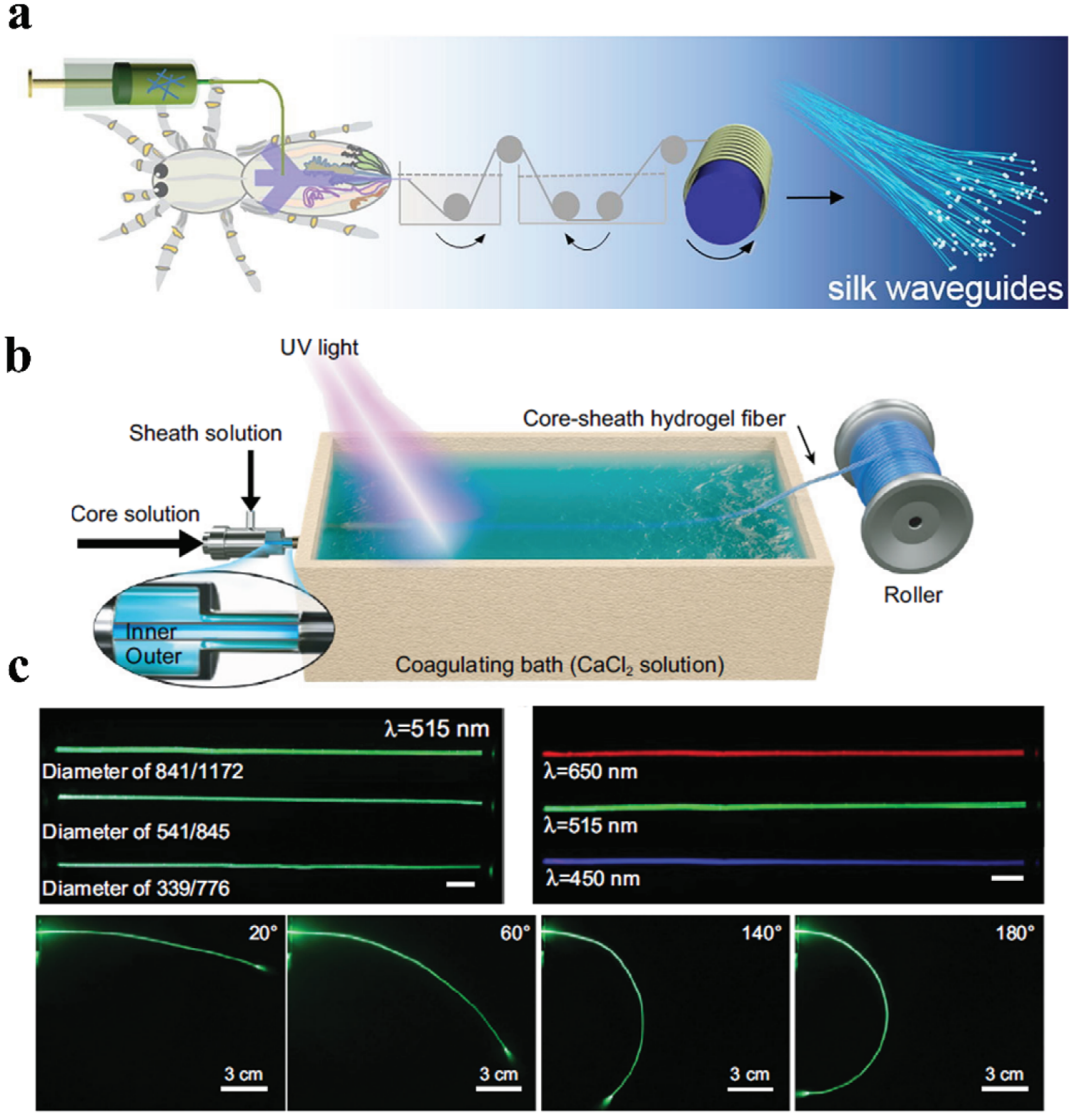
a) Schematic diagram for preparation of light waveguide fibers by wet‐spinning employing a microfluidic chip. Reproduced with permission.^[^ ^119^ ^]^ Copyright 2021, Elsevier. b) Schematic illustration of the integrated dynamic wet‐spinning apparatus. c) Light transmission within hydrogel fiber with different fiber diameters and different laser wavelengths (scale bar: 1 cm), and photographs of light propagation through the hydrogel fiber with different bending angles. Reproduced under the terms of the Creative Commons CC BY License.^[^ ^85a^ ^]^ Copyright 2020, Oxford University Press.

The Wang group prepared artificial fibers as implantable optical waveguides using genetically engineered spidroin. The artificial fibers were obtained by pipetting the recombinant spidroin solution into polytetrafluoroethylene tubes followed by evaporation of the solvent, exhibiting optical loss of 0.8 ± 0.1 dB cm^−1^ in air and 1.9 ± 0.3 dB cm^−1^ in mouse muscles.^[^ ^118^ ^]^ The Zhang group prepared RSF/CNF hybrid fibers through a microfluidic channel by mimicking the spinning apparatus of spiders, which exhibited a light loss of 1.0 dB cm^−1^ (**Figure** **7**a).^[^ ^119^ ^]^

In addition, hydrogel fibers can be used for light transmission. The Zhu group prepared a core–sheath hydrogel fiber continuously using light‐triggered dynamic wet‐spinning (Figure 7b). In this study, the sheath precursor was a sodium alginate aqueous solution, while the core precursor was a mixture of PEGDA and acrylamide (AAm) aqueous solution. The spinning solution was extruded into the calcium chloride coagulation bath using a core–sheath spinning needle, and the calcium ions diffused into the sodium alginate to form calcium alginate. At the same time, free radical polymerization of PEGDA and AAm was triggered by 360 nm UV light near the spinning nozzle to form the hydrogel network. Figure 7c shows that each visible wavelength efficiently propagated through the core–sheath hydrogel fiber, and the curved fibers could also propagate light. The hydrogel fiber could transmit light in porcine tissue, exhibiting a light loss of 0.62 dB cm^−1^.^[^ ^85a^ ^]^ In another study, a pre‐gel was formed using a precursor solution consisting of clay, oligo(ethylene glycol) methacrylate (OEGMA), and AAm through a certain period of free radical polymerization. The pre‐gel was then extruded to form a fiber and was further post‐stretched to form a highly oriented hydrogel fiber. The resulting hydrogel fiber exhibited a light propagation attenuation of 0.26 dB cm^−1^.^[^ ^120^ ^]^ Because of their good biocompatibility, hydrogel fibers are promising for various potential applications such as photomedicine, optical biosensing, and brain‐machine interfaces.

### 3.4 Torsional Energy Dissipation and Shape‐Memory Behavior

#### 3.4.1 Torsional Energy Dissipation and Shape‐Memory Behavior of the Spider Silk

When in danger, the spider escapes by hanging on the dragline silk, which does not swing around the initial position and never rotates back and forth when it is torsionally triggered. This stability is conducive to the concealment of hanging spiders and reduces the probability of being spotted by predators.^[^ ^5^, ^121^ ^]^ Studies have also shown that spider dragline silk exhibits peculiar shape‐memory behavior together with an energy damping capacity.^[^ ^121a^ ^]^ Emile et al. studied the torsional damping properties of Kevlar thread, copper wire, spider dragline silk, and shape‐memory Nitinol alloy wires. After suspending a certain weight and being twisted by 90°, these threads were released, and the torsional behavior was characterized by a camera. Kevlar was untwisted to the starting position and oscillated for a period of time after releasing the twist, which showed little energy dissipation. The copper wire deformed after torsion, did not recover its initial position after torsional deformation, and rotated around the new equilibrium position, which showed a high damping capacity. The spider dragline silk also exhibited a high damping capacity. In contrast to copper wire, dragline silk can completely recover its initial position after torsion in the subsequent 6000 s in an exponential manner. This is similar to the shape‐memory behavior observed for the shape‐memory NiTi wire. The difference is that spider silk can return to its original position without other excitations (e.g., heating), while the NiTi wire can only recover its original position when it is heated to 90 °C.

The high torsional damping capacity of spider dragline silk was further systematically studied by the He group, who ascribed the high energy dissipation to its hierarchical structure, including the microscopic canaliculi and nanostructures. The *β*‐sheet nanocrystals act as the nodes to maintain the permanent shape during torsion because of their high mechanical stiffness, and the highly coiled and disordered molecular chains in the amorphous region can absorb and dissipate the input energy during the initial twist insertion.^[^ ^5^ ^]^ In this study, the shape‐memory effect of the dragline silk was not observed, which could be ascribed to the differences in the spider species, and environmental and other testing conditions. Therefore, further investigation is needed for an in‐depth study of shape‐memory behavior and the underlying mechanism in different scenarios.

The spider silk after supercontraction can be re‐stretched to its original length, which can again perform supercontraction upon exposure to high humidity.^[^ ^122^ ^]^ The humidity‐responsive supercontraction behavior of spider silk is a typical shape‐memory behavior.^[^ ^123^ ^]^

#### 3.4.2 Artificial Fibers Exhibiting Torsional Energy Dissipation and Shape‐Memory Behavior

Inspired by the shape‐memory function of spider silk, the Hu group intensively studied bio‐inspired shape‐memory fibers from recombinant MaSp2 by wet‐spinning.^[^ ^123^, ^124^ ^]^ This fiber was stretched to a specific strain at 20% RH and held for 10 min while maintaining the strain. After removing the tethering, the stretched fiber remained at this length, which recovered to its original length upon exposure to 75% RH, exhibiting a shape fixity of 82.1 ± 2.1% and recovery ratio of 98.5% ± 0.4%.^[^ ^124a^ ^]^ The mechanism underlying such shape‐memory behavior is that the *β*‐sheet crystals within the artificial fiber act as net points to stabilize their permanent shape, and the H‐bonds are designated as switches in the amorphous region to trigger shape deformation and recovery. In two separate studies, short‐chain polyalanine (net points) was introduced into multiblock biopolymers containing poly(ɛ‐caprolactone) segments (*β*‐sheet crystals) via a coupling reaction; nearly complete shape recovery and high shape fixity was observed.^[^ ^123^, ^125^ ^]^

The hybrid silk material fabricated by introducing silk fibroin into poly(vinyl alcohol) (PVA), has a two‐way shape‐memory effect because of the competitive H‐bonds between silk fibroin and PVA from water as a switch, the *β*‐sheet structure as net points, and the swelling plasticizing effect of its asymmetric laminated structure.^[^ ^124b^ ^]^ Polyurethanes contain soft and hard segments, which are similar to the crystalline and amorphous regions of spider silk. The Li group prepared a polyurethane fiber by melt‐spinning, which was annealed at 100% strain at 100 °C for 1 h to thermally set the shape. The resulting fiber recovered its initial shape by reheating to 100 °C.^[^ ^126^ ^]^

Spider silk exhibit a high damping capacity to eliminate rotation when the hanging spider was torsionally triggered.^[^ ^127^ ^]^ This damping effect of the spider silk can be attributed to the coordination of the crystalline (alanine block) and the amorphous blocks (glycine block), where the crystalline block provides rigidity and the amorphous block mainly dissipates rotating energy.^[^ ^5^ ^]^ Inspired by such torsional mechanical damping behavior, the Kim group prepared a torsional fiber actuator exhibiting a high torsional damping capacity by employing a twisted CNT yarn that was imbued with paraffin wax and polystyrene‐poly(ethylene‐butylene)‐polystyrene (SEBS) copolymer. Upon heating, the torsional artificial muscle generated ultrafast rotation, and the muscle exhibited an overdamped dynamic response after removing the stimulus; a damping time that was tenfold less than that of a twisted CNT yarn was observed.^[^ ^128^ ^]^

### 3.5 Thermal Conductance of the Spider Silk

The crystalline region of spider silk is mainly composed of highly oriented, antiparallel *β*‐pleated sheets. The Wang group found that spider dragline silk exhibited higher thermal conductivity than most materials, including some good thermal conductors such as silicon, aluminum, and iron.^[^ ^3^ ^]^ The thermal conductivity of the individual spider silk and its strain‐dependence were measured using a customized electrothermal technique, where a spider silk was glued by employing silver paste for thermal conductivity measurement. The thermal conductivity of the spider silk increased as the strain increased, reaching a maximum value of 416 W m^−1^ K^−1^ at a strain of 19.7%. The high thermal conductivity of spider silk was ascribed to the spidroin structure, such as the highly conserved repetitive sequences and non‐repetitive CTD and NTD.^[^ ^129^ ^]^ The highly aligned molecular chains in the axial direction of the spider silk with negligible defects also contributed to the high thermal conductivity. During strain increase, the axial orientation of the secondary structures was improved, consequently resulting in increased thermal conductivity. In order to further explore the influence of the internal structure on thermal conductivity by atomistic simulation and photonic analysis, the Liu group revealed that inter‐*β*‐strand H‐bonding greatly contributed to the thermal conductivity of the spider silk.^[^ ^130^ ^]^ In an anti‐parallel *β*‐sheet composed of five *β*‐strands, where each *β*‐strand has 16 alanine residues, the thermal conductivity of spider silk increases with an increase in the number of *β*‐strands. The high thermal conductivity of spider silk, combined with its robust mechanical properties and biocompatibility, makes it a promising fiber material for heat dissipation in electronics, chips, and especially in epidermal wearable electronics. The study of structural–thermal conductivity would also inspire new design strategies for organic and polymer thermal conducting materials. However, in the current research, the thermal conductivity is generally increased by manipulating the structure of the organic, inorganic, and metal materials and their composites. To the best of our knowledge, despite numerous studies on thermally conducting materials, few studies have correlated the high thermal conductance to the structure of spider silk.

## 4 Conclusions and Perspectives

Spider dragline silk exhibits high strength and toughness, good thermal conductivity, optical properties, supercontraction and torsion, a high damping capacity, and water collection. These unique properties would enable potential applications in energy dissipation, impact absorption, heat dissipation, and in devices such as optical sensors, artificial muscles, soft robotics, and biomedical applications. Understanding the structural mechanisms underlying these properties is important for unraveling the mystery of spider silk. Structural studies reveal that the excellent combination of strength and toughness of the spider silk originates from its hierarchical structure, from the micron to the molecular level. Spider silk is composed of a skin–core structure, where the core is composed of a “soft inner core” and a “rigid outer core”, which dominates its mechanical properties. The core contained highly originated, twisted nanofibers assembled from molecular chains. In these nano‐assemblies, the high‐density H‐bond regions form *β*‐sheet nanocrystals embedded in the soft amorphous regions, which serve as physical cross‐links similar to a “fishing‐net.” These studies provide insight into the design of artificial materials with high strength and toughness. For an in‐depth understanding of the structural mechanisms of these mechanical properties, additional studies are required.

It is difficult to obtain a large quantity of natural spider silk because spiders are aggressive and tend to kill each other if they are raised in a limited space. Therefore, artificially mimicking the internal structure of natural spider silk by employing chemical synthesis, polymer composition, and structural control is highly desirable to obtain spider silk‐like artificial fibers. To date, several different approaches have been proven to be effective in preparing strong and tough fibers by employing recombinant spidroins, non‐spidroin proteins, polymer materials (hydrogels, polyurethanes, cellulose), and their composites. Although great progress has been made, there is still a large gap between the mechanical performances of artificial fibers with natural spider dragline silk because of the difficulty in fully mimicking the complex hierarchical structures of the spider silk. In addition, the spinning of artificial fibers by mimicking the spider ampulla remains a challenge. Currently, there has been some important progress in the development of novel spinning methods, such as draw‐spinning, wet‐spinning, and dynamic cross‐linking spinning. To realize the hierarchical structure from the molecular level, nanometer level, to the micron‐scale level, by employing an energy‐efficient spinning method, is still an issue that needs to be addressed.

The supercontraction behavior of spider silk enables the spider web to tighten or recover its initial shape in the rain or dew after it is deformed by the flying insects. Such a self‐repairing capacity is a type of shape‐memory behavior that can be employed in the design of smart materials. In addition, spider silk exhibits moisture‐driven torsion, contraction, and supercontraction, which can be used in the fields of soft robotics, artificial muscles, and sensors. Furthermore, spider silk exhibits a high damping capacity for energy absorption, which can reduce the oscillation when there is a disturbance of the hanging spider. Realizing these interesting properties by mimicking the structure of spider silk would provide new strategies for the preparation of a variety of smart fiber materials. In addition, spider silk has other interesting properties, including high thermal conductance, good optical transmittance, and biocompatibility. To emulate additional properties (e.g., shape‐memory, self‐repair, or damping capacity) in strong and tough fibers remains a challenge and requires further research. Moreover, by integration with electrical, optical, and magnetic properties, spider silk‐like fibers have promising applications in the fields of wearable electronics, optical sensors, intelligent control, and energy harvesting. These possibilities for material design with multiple functionalities would enable enormous opportunities for further applications in intelligent society.

## Conflict of Interest

The authors declare no conflict of interest.
